# How many tricks can an old perovskite play?

**DOI:** 10.1107/S2052252517005656

**Published:** 2017-04-25

**Authors:** Brendan J. Kennedy

**Affiliations:** a School of Chemistry, The University of Sydney, Sydney, NSW, Australia

**Keywords:** perovskite, ferroelectric, powder neutron diffraction

## Abstract

The ferroelectric–paraelectric transition in Li_0.2_Na_0.8_NbO_3_ is between two extremely rare perovskite polytypes. How the unprecedented sequence of structures was established shows that even old oxides can play new tricks.

NaNbO_3_ has long been an enigma amongst the compositionally simple perovskites and elucidating the temperature dependence of its structure was a significant crystallographic achievement (Sakowski-Cowley *et al.*, 1969 ▸; Peel *et al.*, 2012 ▸). But having climbed that mountain, Lightfoot and co-workers (Dixon *et al.*, 2017 ▸) describe the next challenge – what does chemical doping do to the structure of NaNbO_3_? To address this they have studied the Li-doped perovskite Li_0.2_Na_0.8_NbO_3_. Their aim is to understand the structural origin of the piezoelectric and electro-optic properties of the alkali-earth-containing niobates. The goal of society is to replace PZT Pb(Zr,Ti)O_3_ with a lead-free material, the prize being a share of the multibillion dollar piezoelectric market (Rödel *et al.*, 2009 ▸).

The first step in the crystallographic analysis was to choose a technique that allowed Lightfoot and co-workers to determine the thermal evolution of the phase behaviour and crystal structure of Li_0.2_Na_0.8_NbO_3_. For this they turned to high-resolution powder neutron diffraction; powder diffraction eliminates artefacts due to twinning and neutrons provide sensitivity to displacement of the anions and lighter cations. Then it was necessary to understand the growth and disappearance of weak ‘superlattice’ reflections associated with the crystallographic phase transitions. For that symmetry-mode analysis was indispensable and was achieved with the *ISODISTORT* suite. It should go without saying that the sample needs to be ‘suitable’, that is free of impurities and highly crystalline, and the authors achieved this goal. The quality of the experimental data presented leaves nowhere for ‘bonus’ peaks to hide.

Having settled on a strategy, what results is a masterclass in perovskite crystallography and an elegant demonstration that not all perovskites are the same.

The phase transition sequence observed in Li_0.2_Na_0.8_NbO_3_ (the 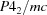 and 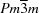 structures are shown in Fig. 1 ▸) is unique in perovskite crystallography. It is also bizarre!
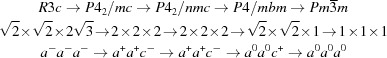
In the ‘average’ perovskite with a low tolerance factor tilts grow or change direction, but rarely do they simultaneously change direction and sense. The octahedral tilts in perovskites arise because the effective radius of the *A*-site cation is smaller than the available volume. The octahedra then rotate to reduce the size of the cuboctahedral interstices in the oxygen sublattice. This tilting is in competition with the displacement of cations. Consider the room temperature *R*3*c* structure (Peel *et al.*, 2013 ▸) then this is described as a 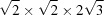 supercell with an 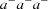 tilt system, using the Glazer tilt notation (Glazer, 1972 ▸). That is, the NbO_6_ octahedra are tilted out of phase with each other relative to each of the three axes of the parent cubic cell, and this causes an increase in the parent cell length. In transforming to the high-temperature non-centrosymmetric 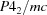 cell the change in tilting results in a change in the unit-cell parameters. In the ‘average’ perovskite tilts grow and change direction and this is evident in the 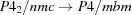 transition where the in-phase or + tilt moves from about [110] to about [001] and the out-of-phase or – tilt is lost. Rarely do tilts simultaneously change direction and sense but this is what happens at the 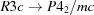 transition where the out-of-phase tilt moves from about [111] to [001] and simultaneously reverses sense to [110], such that one tilt becomes two! The ferroelectric–paraelectric transition in Li_0.2_Na_0.8_NbO_3_ is between two extremely rare perovskite polytypes 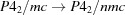 having the same tile pattern.

Unfortunately, the terms elegant and bizarre are often insufficient to excite funding agencies. Nevertheless, the crystallographic results show how piezoelectricity can occur in Li_0.2_Na_0.8_NbO_3_. The next challenge is to establish if, and how, the unusual sequence of structures impacts on the physical properties of the alkali metal niobate perovskites in general and to utilize this insight to generate environmentally benign, commercially viable products that can be utilized in devices such as sensors and actuators. I suspect that before this happens the niobate perovskites have a few more surprises to reveal.

## Figures and Tables

**Figure 1:**
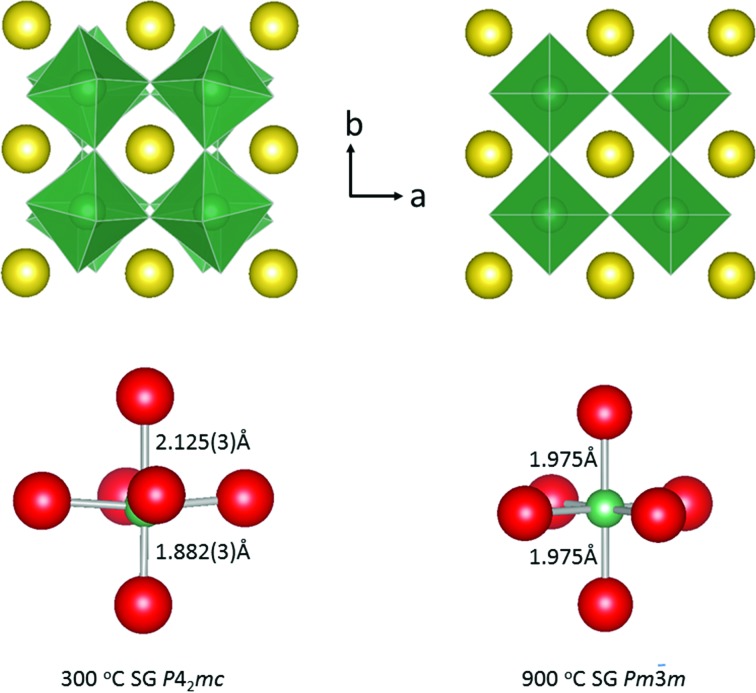
The 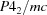 and 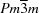 structures at 300 and 900°C, respectively.
